# Mapping the landscape: a comprehensive bibliometric analysis of transversus abdominis plane block research (2007–2024)

**DOI:** 10.1097/JS9.0000000000003312

**Published:** 2025-08-27

**Authors:** Zhiyi Cheng, Can Jiang, Cheng Xu, Miao Wang, Jun Yao, Ni Wenzong, Jie Lu, Aizhong Wang

**Affiliations:** Department of Anaesthesiology, Shanghai Sixth People’s Hospital Affiliated to Shanghai Jiao Tong University School of Medicine, Shanghai, China

## Introduction

Abdominal surgery is the most frequently performed procedure, involving various intra-abdominal and retroperitoneal structures, including the gastrointestinal tract, hepatobiliary system, spleen, kidneys, uterus, and bladder. In the United States, approximately 15 million major inpatient surgeries are performed annually, with over 4 million involving the abdomen^[1]^. Although the advent of laparoscopy has significantly reduced complications associated with open surgeries, most patients still experience moderate-to-severe pain during the perioperative period. Acute postoperative pain not only exacerbates patient discomfort but also complicates perioperative pain management and may impede long-term functional recovery. Consequently, effective and reliable perioperative analgesia for abdominal surgery remains a significant clinical challenge.

Multimodal analgesia has emerged as the gold standard for postoperative pain management following major abdominal surgery. This approach integrates opioids, nonsteroidal anti-inflammatory drugs (NSAIDs), acetaminophen, adjuvant agents, and abdominal-wall blocks to optimize pain management^[2]^. Among regional anesthesia techniques, abdominal-wall nerve blocks have garnered increasing attention. The transversus abdominis plane block (TAP-B) has become a widely adopted modality for perioperative analgesia in both laparoscopic and open abdominal procedures. When integrated into multimodal regimens, TAP-B has demonstrated potential benefits in reducing opioid requirements and expediting postoperative recovery^[1,2]^. However, the analgesic efficacy of TAP-B remains a subject of ongoing debate due to inconsistent clinical outcomes, conflicting data regarding its effect on functional recovery, and block-related adverse effects. Therefore, further well-designed, comparative studies are required to delineate the specific indications, optimize block techniques, and evaluate the relative efficacy of TAP-B against other emerging regional anesthetic modalities.

Bibliometrics provides a systematic approach to quantitatively analyze research patterns within scientific literature^[3]^. Since its inception in 1955 with Eugene Garfield’s Science Citation Index, bibliometrics has evolved into a robust tool for identifying research trends, shifts in therapeutic approach, disease patterns, and system-level concerns in medicine. Advanced software such as CiteSpace, VOSviewer, and Bibliometrix enhances the visualization and interpretation of publication trends^[3]^. Although bibliometrics has gained traction in regional anesthesia, dedicated analyses focusing on TAP-B remain limited, underscoring the need for a comprehensive bibliometric assessment of TAP-B research to characterize its evolution, identify research hotspots and knowledge gaps, and inform future investigations within the field.

This study systematically analyzed TAP-B-related publications from 2007 to 2024, providing an evidence-based overview of the field’s development, recent advancements, and emerging research frontiers. All analyses were conducted according to the 2025 TITAN AI-reporting standards^[4]^.

## Methods

### Data source and search strategy

Data for this bibliometric analysis were obtained from the well-curated Web of Science Core Collection (WoSCC) citation index. A systematic search was conducted on 31 May 2024, covering the publication period from 1 January 2007 to 1 May 2024. The search strategy, guided by Medical Subject Headings (MeSH), employed the following topic search (TS) query: “transversus abdominis plane” OR “TAP-B” OR “TAP block.” To preserve data integrity and prevent incorporation of future database updates, the dataset was locked on the date of retrieval. The initial search yielded 1702 English-language articles. Only original research articles were included; editorial letters, commentaries, reviews, brief reports, retracted publications, and duplicates were excluded. As the study involved analysis of publicly available literature without the inclusion of human subjects or patient data, ethical approval was not required.

### Study selection and data management

Following data retrieval, all records were imported into CiteSpace (version 6.3 R1) to detect and eliminate duplicates and reduce manual processing errors. No redundant entries were identified. To ensure consistency in keyword-based analyses, synonymous terms as well as singular and plural forms were harmonized. Data extraction, processing, and standardization were performed independently by three reviewers. The results were cross-validated, and discrepancies were resolved through consensus involving a third reviewer. The final dataset comprised 702 eligible original articles. Statistical adequacy of the final dataset was confirmed through comparisons with prior bibliometric studies in related fields.

### Data analysis

Bibliometric analysis of TAP-B literature was performed using three complementary tools: CiteSpace 6.3 R1, VOSviewer 1.6.18, and Bibliometrix 4.1. CiteSpace was utilized for its ability to identify influential publications, track citation bursts, and detect emerging research frontiers through visual analytics. VOSviewer was employed to construct co-authorship, institutional, and country collaboration networks, as well as co-citation maps, leveraging its capability for processing large bibliometric datasets. Bibliometrix, an open-source R suite, facilitated bibliometric workflows. The Bibliometric.com portal leverages this package for mapping and visualization.

The bibliometric indicators analyzed in this study included authors, countries, institutions, journals, cited references, and keyword distributions. VOSviewer was specifically used to generate co-occurrence and clustering maps for authors and institutions, where nodes represented entities, and cluster colors denoted thematic groupings. CiteSpace was used to construct dual-map overlays of citing and cited journals, citation bursts, and keyword clusters. Clusters were automatically labeled based on title words, keywords, and abstract terms from citing articles. Bibliometrix generated additional outputs, including publication trends by institution and author, keyword frequency distributions, and heat maps. SCImago Graphica 1.0.35 and Microsoft Excel 2021 were used to produce supplementary statistical visualizations. Journal impact factors were obtained from the 2024 edition of the WoSCC Journal Citation Reports to assess journal influence and quality.

## Results

The research process is outlined in the flowchart presented in Supplemental Digital Content Figure S1, available at: http://links.lww.com/JS9/E961. The initial search of the WoSCC database identified 1702 English-language articles. Following application of the inclusion and exclusion criteria, a total of 702 publications were deemed eligible for comprehensive bibliometric analysis.

### Annual publication volume and trends

**Figure 1.**
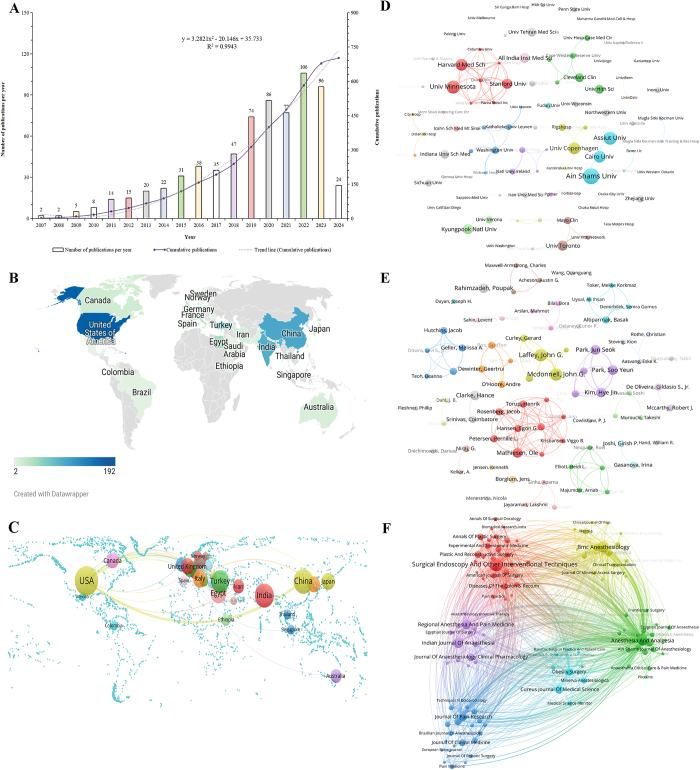
(A) Cumulative number of TAP-B-related publications per year. (B) Global geographic distribution of TAP-B publications. (C) Country-level co-authorship network map. (D) Institutional co-authorship network map. (E) Author-level co-authorship network map. (F) Journal co-citation network map. *Note:* For interpretation of color coding in this figure, please refer to the web version of this article. TAP-B, transversus abdominis plane block.

Figure 1A shows both the annual output and the cumulative growth of TAP-B-related literature. From 2007 to 2010, research activity in this field was limited, with fewer than 30 articles published annually. A significant upward trend was observed after 2014, with the number of publications rising from 35 in 2017 to a peak of 106 in 2022, reflecting a threefold increase over 5 years. A binomial regression model (*R*^2^ = 0.9943) confirmed a statistically significant increase in publication output over the 18-year study period, indicating the growing prominence of TAP-B as a research hotspot in anesthesiology and pain management.

### Geographic and institutional contributions

A total of 947 institutions from 53 countries contributed to the TAP-B literature (Fig. 1B). The 10 most productive countries are outlined in Supplemental Digital Content Table S1, available at: http://links.lww.com/JS9/E961. The United States ranked first with 192 publications, representing over 25% of the total research output, followed by China (98) and India (91). When evaluated by mean citation count, the United Kingdom (52.3), Canada (30.4), and Japan (16.9) exhibited the highest averages, reflecting greater impact. In contrast, China’s lower citation rate despite its high publication output suggests the need to enhance the methodological rigor and global influence of its contributions. The top 10 publishing countries formed a tightly connected collaboration network, with the United States serving as a central anchor for these international collaborations, while China, Germany, and Turkey were also prominent within this network (Fig. 1C). These findings highlight the importance of transnational partnerships in advancing the field.

At the institutional level, the University of Copenhagen emerged as the most prolific contributor with 11 publications (Supplemental Digital Content Table S2, available at: http://links.lww.com/JS9/E961). Other notable institutions included Duke University, Harvard University, Pacira Biosciences, Stanford University, and West Virginia University (Fig. 1D). These institutions represent major hubs of TAP-B research, with strong inter-institutional collaborations observed, particularly among institutions in the United States, Australia, and Europe. Despite these regional partnerships, country-level data indicated that overall global collaboration remains limited, underscoring the need for more inclusive and geographically diverse partnerships to advance TAP-B research.

### Analysis of authorship and collaborative networks

A total of 3919 authors contributed to the 702 TAP-B publications analyzed in this study. Among these authors, Laffey JG and McDonnell JG, both affiliated with the National University of Ireland, were identified as the most prolific contributors, each authoring five original research articles within the field. McDonnell JG also emerged as the most cited author, with a cumulative citation count of 1531, reflecting significant academic influence and foundational contributions to TAP-B research. Figure 1E depicts the author collaboration network. Mathiesen Ole demonstrated the most extensive collaborative relationships, underscoring a key role in fostering partnerships and advancing collective research efforts within TAP-B research.

### Source and co-cited journal analysis

Journal distribution patterns were evaluated using VOSviewer. The results revealed that the 702 TAP-B articles were published across 221 peer-reviewed journals. *Surgical Endoscopy and Other Interventional Techniques* emerged as the leading source, contributing 32 articles (4.6%), followed by *BMC Anesthesiology* with 24 publications (3.4%). Among the top 10 journals, *Regional Anesthesia & Pain Medicine* demonstrated the highest impact factor (5.1), reflecting its significant influence and field relevance. Notably, 40% of the top 10 journals were indexed within the Q1 quartile (Supplemental Digital Content Table S3, available at: http://links.lww.com/JS9/E961), indicating high scientific impact. The co-citation network of these journals is presented in Figure 1F. *Anesthesia & Analgesia* (606 citations), *British Journal of Anaesthesia* (353 citations), and *Surgical Endoscopy* (305 citations) were identified as the most co-cited journals, indicating their central role in TAP-B research development.

**Figure 2.**
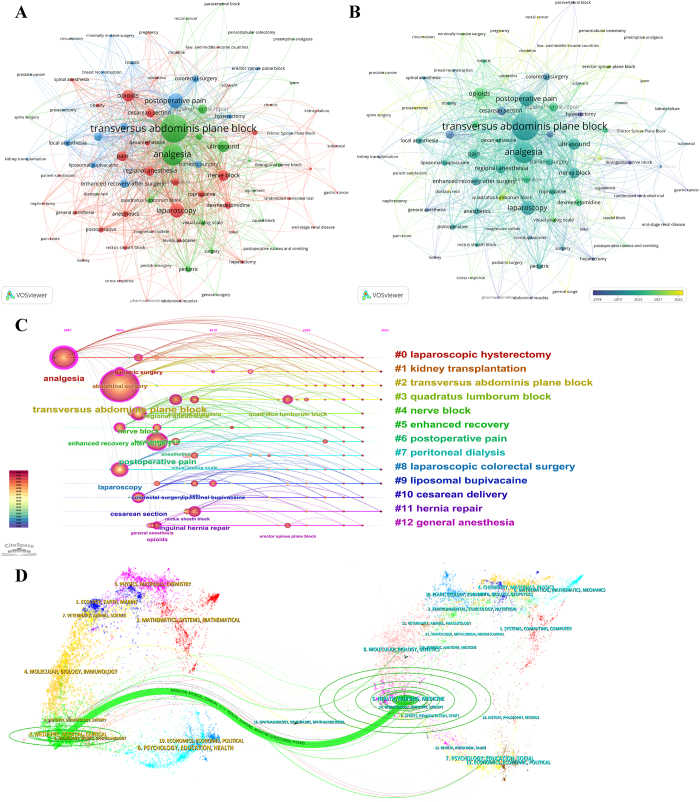
(A) Network map of keyword co-occurrence. (B) Overlay visualization of keyword co-occurrence. (C) Timeline visualization of keyword co-occurrence. (D) Dual-map overlay of citing (left) and cited (right) journals related to TAP-B research; citation relationships are indicated by colored paths. TAP-B, transversus abdominis plane block.

A dual-map overlay (Fig. 2D) was used to illustrate the citation dynamics between citing (left) and cited (right) journals through colored paths. The most prominent citation trajectory (denoted by the orange path) revealed that articles published in medicine and clinical journals most often cite literature from health, nursing, and medicine journals, indicating a strong interdisciplinary relevance of TAP-B research.

### Analysis of highly cited studies

The 10 most-cited TAP-B studies are listed in Supplemental Digital Content Table S4, available at: http://links.lww.com/JS9/E961. These influential publications originated from diverse regions: four from Europe, three from Asia, two from Oceania, and one from Africa. The most-cited article was a 2016 study titled “Quadratus Lumborum Block Versus Transversus Abdominis Plane Block for Postoperative Pain After Cesarean Delivery: A Randomized Controlled Trial,” published in *Regional Anesthesia & Pain Medicine*, with 67 citations.

Among the top-cited articles, four evaluated the efficacy of TAP-B in comparison with other regional analgesic techniques such as quadratus lumborum block, ilioinguinal/iliohypogastric block, and epidural analgesia. Three studies investigated the pharmacodynamics of TAP-B, including serum anesthetic concentrations, spread patterns, as well as the synergistic effects of adjuvants such as dexmedetomidine. The remaining three articles explored the anatomical basis of ultrasound-guided TAP-B, its spread patterns, and performance in bariatric surgery. Citation-burst analysis (Supplemental Digital Content Figure S2, available at: http://links.lww.com/JS9/E961) was performed to identify high-impact publications over time. The 2007 TAP-B randomized controlled trial by McDonnell JG exhibited the highest citation burst (strength 18.95). Other sustained citation bursts included McDonnell (2007, 2008) and Tran TMN (2009), all persisting for more than 5 years. These patterns highlight the foundational and sustained influence of these studies in shaping TAP-B practice and scholarship.

### Keyword analysis of research hotspots

Keyword co-occurrence mapping was conducted to identify major research themes and emerging trends. These networks are depicted in Figure 2A and B through clear overlay maps. The 10 most frequent keywords included TAP block, analgesia, postoperative pain, laparoscopy, opioids, ultrasound, nerve block, pain, regional anesthesia, and hernia repair. Figure 2A categorizes these keywords into three primary clusters. The green cluster encompasses terms related to TAP-B analgesic techniques such as analgesia, ultrasound, anesthesia, and quadratus lumborum block. The blue cluster highlights surgical applications, including abdominal surgery, bariatric surgery, colorectal surgery, and hysterectomy. The red cluster highlights terms related to TAP-B pharmacology, such as opioids, pain, regional anesthesia, and bupivacaine. Figure 2B depicts temporal trends in keyword usage. Purple-colored terms represent keywords that were predominant before or around 2018, while bright-yellow terms denote terms that gained prominence after 2022. Early studies primarily focused on “abdominal surgery” and “cesarean section,” whereas terms such as “quadratus lumborum block” and “erector spinae plane block” emerged only in recent studies, reflecting the field’s recent pivot toward comparative fascial plane blocks and newer interfascial techniques.

To assess the chronological evolution of research foci, a time-slice keyword analysis was conducted using CiteSpace (Fig. 2C). The resulting map yielded a modularity (Q) score of 0.83 and a silhouette (S) score of 0.96, indicating strong and coherent clustering. Before 2015, dominant research themes included TAP-B and laparoscopic hysterectomy. By 2024, research focus had shifted toward enhanced recovery protocols, comparative analysis between TAP-B and quadratus lumborum block, use of liposomal bupivacaine, and laparoscopic colorectal surgery.

## Discussion

This bibliometric analysis revealed a significant and sustained increase in both publication volume and citations of TAP-B literature between 2007 and 2024. The early years of this period were marked by modest activity; however, a notable acceleration in publication output was observed as ultrasound-guided regional anesthesia techniques became more refined and accessible. Between 2017 and 2022, the annual number of publications rose from 35 to 106, reflecting growing clinical adoption of TAP-B and its integration into multimodal analgesia protocols for abdominal surgery. This upward trend parallels advancements in medical and technical modalities, which have positioned TAP-B as a foundational component of abdominal multimodal analgesia. Given these developments, the expansion of TAP-B research is anticipated to persist, contributing to global advancements in analgesic strategies and perioperative care.

### Basic information analysis

Analysis of basic publication information identified the United States as the leading contributor to TAP-B research in both volume and influence, followed by China and India. This leadership reflects significant governmental and institutional investments in scientific and healthcare research and development, which inherently fosters innovation. The United States, the United Kingdom, and Turkey emerged as the top three countries by total citations. Notably, the United Kingdom exhibited the highest mean citations per article, underscoring the depth and scholarly impact of its research. Conversely, China, despite ranking second in total publications, was fourth in total citations and 10th in average citations per article. These findings suggest that while research productivity is high, the lower citation rates warrant improvements in study design, methodological rigor, and reporting transparency to enhance the scholarly impact of Chinese TAP-B studies. Collaborative mapping further revealed that the United States remains the central hub of international TAP-B research networks, although China’s presence is rapidly growing. Elevating the scholarly quality of Chinese TAP-B studies through focused research training, meticulous study design, and disciplined execution is essential for advancement in this field. Furthermore, increased funding, access to modern research equipment, and international collaboration can significantly increase the scientific value of TAP-B studies originating from China. Additionally, academic evaluation systems that prioritize intellectual insight over publication quantity may help incentivize high-quality research and increase China’s influence in the global academic discourse on TAP-B.

Authorship trends revealed dense collaborative networks, with key contributions from researchers such as Laffey JG and McDonnell JG (both affiliated with the National University of Ireland), who have authored numerous randomized controlled trials investigating abdominal regional anesthesia and studies mapping anesthetic spread patterns following TAP-B. McDonnell JG exhibited the highest citation count, largely attributable to his pioneering randomized trials, positioning him as a thought leader in the field. Journal co-citation analysis identified *Anesthesia & Analgesia* as the most cited sources, followed by the *British Journal of Anaesthesia*. These findings reinforce their status as core publications in anesthesiology. The most cited study was a 2016 randomized controlled trial by Rafael Blanco, which compared quadratus lumborum block and TAP-B for post-cesarean analgesia (Supplemental Digital Content Table S4, available at: http://links.lww.com/JS9/E961). The quadratus lumborum block demonstrated superior analgesic efficacy compared with TAP-B, as evidenced by a reduction in postoperative morphine requirements. These findings reignited scientific debate regarding the precise mechanisms and overall clinical efficacy of TAP-B, prompting further comparative and anatomical investigations.

Keyword co-occurrence mapping serves as an invaluable tool for identifying disciplinary structure and emerging research themes within TAP-B literature. When integrated with citation analysis, this approach can effectively track shifts in academic focus over time. In this study, “analgesia” remained the most frequent keyword in TAP-B inquiry, highlighting its persistent relevance as the primary research objective in TAP-B research. Researchers continue to scrutinize TAP-B pain-relief performance. This emphasis is consistent with findings from recent systematic reviews. A meta-analysis in adults reported a statistically significant, albeit clinically modest, reduction in postoperative morphine requirement with TAP-B for abdominal surgery – approximately 6 mg at 6 h and 11 mg at 24 h postoperatively^[5]^. In pediatric populations, evidence suggests that TAP-B provides short-term analgesic benefit limited to the immediate postoperative period^[6]^. Both reviews highlight significant heterogeneity in the study population and outcome measures, which limits the generalizability of current findings and highlights the need for standardized methodologies and consensus definitions to accurately delineate TAP-B’s role within modern perioperative analgesia protocols.

### Theme trends and topics of interest

Variations in TAP-B techniques result in distinct anesthetic spread patterns, which significantly influence analgesic outcomes. Recognizing these anatomical and procedural differences is essential to accurately assess the overall clinical efficacy of TAP-B and to reconcile inconsistencies across published findings. Ultrasound-guided TAP-B follows a unique trajectory of anesthetic spread, unlike landmark-based approaches, offering more reliable deposition of local anesthetic within the transversus abdominis fascial plane^[7]^. Its characteristic posterior-cranial spread into the thoracic paravertebral space is believed to be the underlying mechanism for the superior analgesic effect reported in many clinical studies.

In upper abdominal surgery, the ultrasound subcostal TAP-B generally outperforms the lateral approach due to its more effective coverage of supra-umbilical dermatomes. For complex procedures, a bilateral dual block combining both subcostal and lateral approaches can be employed. When thoracic epidural analgesia is contraindicated, subcostal TAP-B can provide adequate pain relief with fewer side effects, particularly reduced risk of hypotension^[8]^. However, thoracic epidural analgesia remains superior to subcostal TAP-B in cases involving extensive lateral surgical incisions, dominant visceral pain, or in the absence of a continuous TAP catheter to prolong the block duration. Initial reports suggesting benefits of lateral TAP-B in upper abdominal procedures were likely confounded by infra-umbilical port placement and the absence of comprehensive multimodal analgesic regimens. More recent trials indicate only modest reductions in opioid consumption and pain scores with lateral TAP-B^[9]^. Consequently, TAP-B is seldom used in laparoscopic cholecystectomy or bariatric surgery, where systemic multimodal analgesia and local port site infiltration already provide adequate postoperative pain control^[10]^.

In lower abdominal surgery, TAP-B can effectively attenuate early postoperative somatic pain; however, its analgesic benefit is often modest and transient, particularly after administration of NSAIDs and acetaminophen^[11]^. Its limited efficacy in procedures involving significant visceral pain, such as pelvic surgeries, is attributable to the somatic-only coverage of TAP-B. In open inguinal hernia repair, both lateral and posterior TAP-B have demonstrated superiority over no block and local infiltration in reducing pain and opioid requirements^[10]^. Similar results have also been observed following laparoscopic hernia repair^[12]^. Notably, only one hernia study to date has evaluated TAP-B efficacy in combination with a complete multimodal analgesic regimen, including acetaminophen, NSAIDs, and local infiltration^[12]^. Therefore, robust randomized controlled trials are still needed to validate TAP-B’s benefits when comprehensive multimodal analgesia is already routine.

### Future research directions

Despite the growing body of literature, several critical knowledge gaps remain, limiting definitive clinical recommendations for TAP-B use. Priority areas for future investigation include direct comparisons between posterior and subcostal TAP-B techniques in upper abdominal surgery, mechanistic comparisons with lateral quadratus lumborum blocks to better understand differential analgesic pathways, and dose-finding studies to determine optimal local anesthetic volume, technique, and adjuvant use. Posterior TAP-B warrants further evaluation in surgeries where lateral TAP-B has shown suboptimal efficacy, such as laparoscopic appendectomy, laparoscopic hysterectomy, and open prostatectomy. To ensure clinical relevance and reduced bias, future studies must include control groups receiving comprehensive multimodal analgesia.

Given the potential of posterior TAP-B to block sympathetic fibers and provide visceral analgesia, comparative trials against thoracic epidural analgesia in open colorectal surgery should assess not only analgesic efficacy but also secondary outcomes such as pulmonary function, bowel recovery, and hypotension. Furthermore, as the repertoire of ultrasound-guided truncal blocks continues to expand, well-powered studies are warranted to compare TAP-B with newer interfascial plane block techniques, such as erector spinae plane, retrolaminar, and anterior quadratus lumborum blocks. Future randomized controlled trials should incorporate standardized outcome measures, including postoperative pain scores, rescue opioid consumption, cost-effectiveness metrics, and clinically meaningful endpoints, such as length of hospital stay and time to ambulation.

### Limitations

This study has several limitations. First, the bibliometric analysis was confined to the WoSCC, excluding other major databases such as Embase, Scopus, PubMed, and Google Scholar. Although WoSCC includes many high-impact journals, omission of articles indexed exclusively in other databases may have introduced selection bias^[13]^. Second, bibliometrics primarily quantifies research productivity and citation frequency but does not assess methodological quality or clinical relevance of the included studies. Future analyses should incorporate qualitative assessments, such as study design robustness and patient-centered outcomes. Third, citation metrics are influenced by numerous factors – including journal impact, author reputation, and publication language – and may not accurately reflect scientific merit. As such, high-quality studies may be undervalued due to limited citation visibility. Finally, the scope of bibliometric reviews is inherently narrow, focusing on publication outputs and citation patterns, and does not directly assess experimental rigor, reproducibility, or translational applicability of the studies evaluated.

## Conclusion

TAP-B has emerged as a key contemporary regional anesthesia technique in abdominal surgery. Over the past decade, its publication volume has increased 10-fold, providing a robust evidence base and a strong conceptual foundation. Current research activity predominantly clusters around optimizing analgesic efficacy, ultrasound guidance, and anesthetic techniques. Future studies should prioritize delineating fascial spread patterns, evaluating TAP-B efficacy within comprehensive multimodal analgesic regimens, and benchmarking its performance against emerging truncal block alternatives to establish its definitive role in perioperative pain management.

## Supplementary Material

## Data Availability

De-identified data demonstrating the outcomes of this investigation are accessible upon appropriate request from the corresponding author.
