# Supplementary material for: Mapping the landscape: a comprehensive bibliometric analysis of transversus abdominis plane block research (2007–2024)
Source: Int J Surg. 2025 Aug 27;112(1):1865–72. doi: 10.1097/JS9.0000000000003312 (PMC12825844; doi:10.1097/JS9.0000000000003312)
Supplement: Supplementary file 1 [file js9-112-1865-001.docx]

**Supplementary information**

**Mapping the landscape: a comprehensive bibliometric analysis of transversus abdominis plane block research (2007–2024)**

**
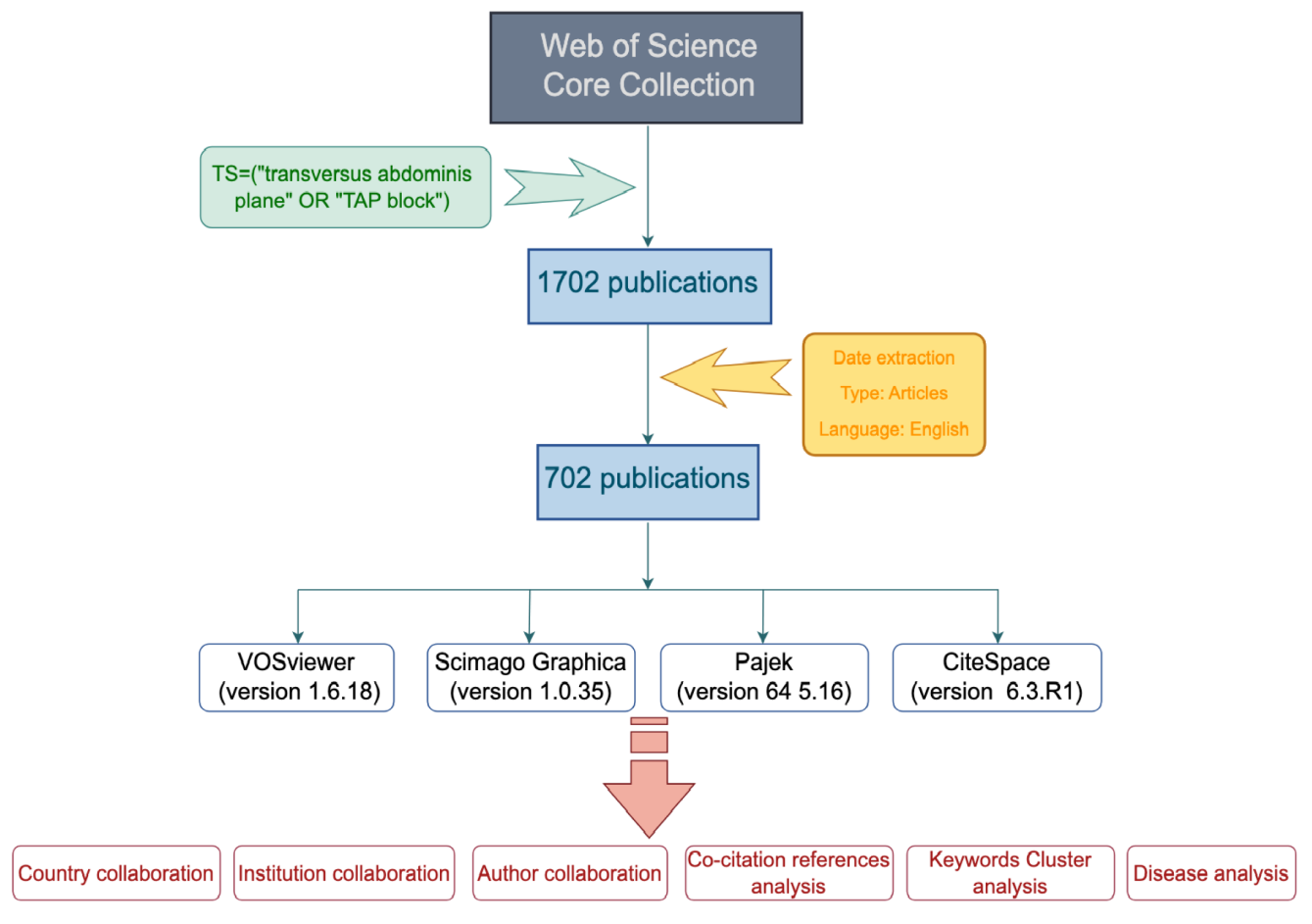
**

**Figure S1.** Flow diagram of the research process. TAP, transversus abdominis plane block.


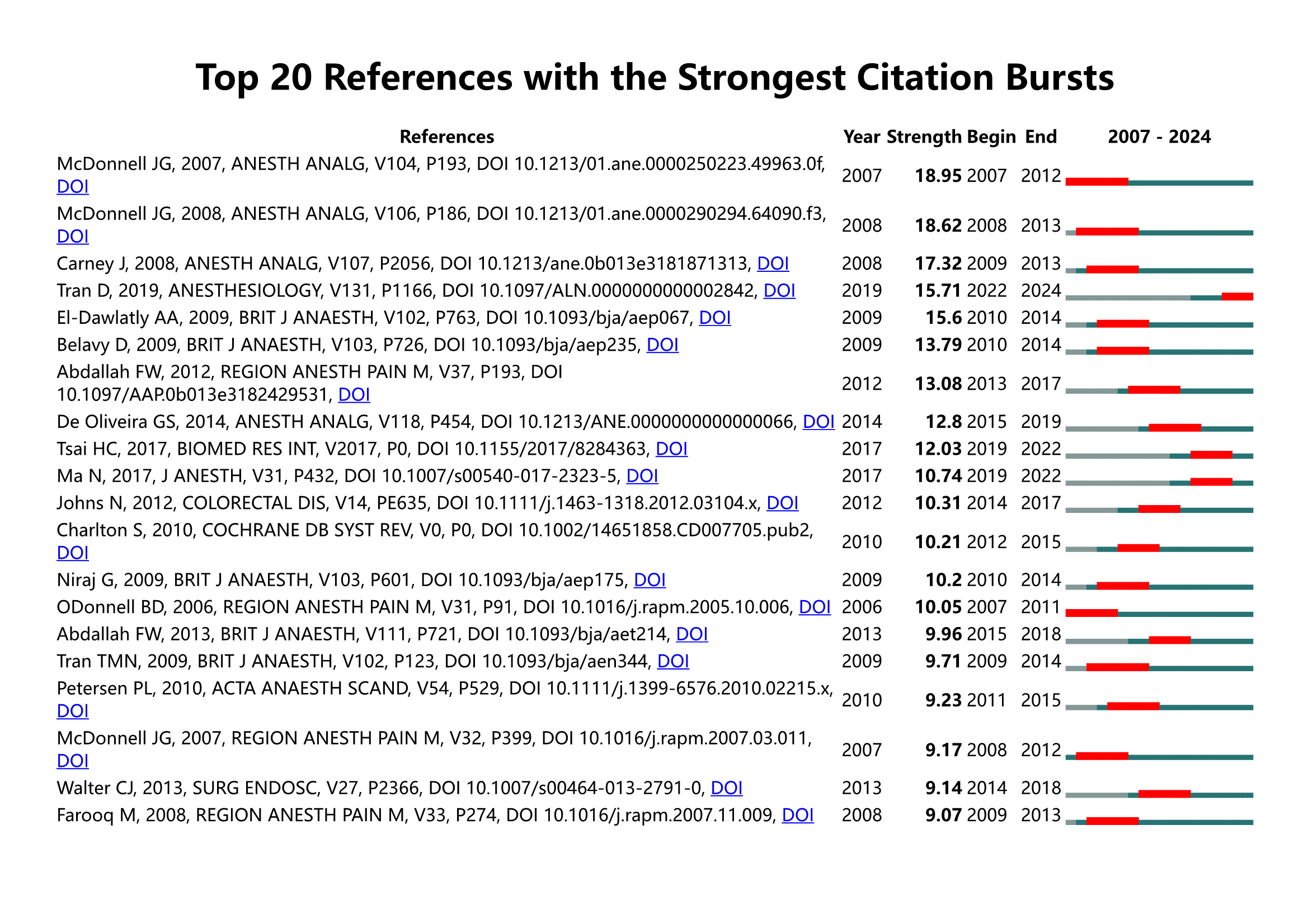


**Figure S2.** Top 20 references with the strongest citation bursts in TAP block research. TAP, transversus abdominis plane block.

**Table S1.** Top 10 countries/regions contributing to TAP block research from 2007 to 2024.

| Rank | Country/Region | Number of articles | Percentage (%) | Total citations | Average citation per article |
| --- | --- | --- | --- | --- | --- |
| 1 | United States | 192 | 27.35 | 2913 | 15.17 |
| 2 | China | 98 | 13.96 | 662 | 6.76 |
| 3 | India | 91 | 12.96 | 660 | 7.25 |
| 4 | Turkey | 70 | 9.97 | 904 | 12.91 |
| 5 | Egypt | 44 | 6.27 | 335 | 7.61 |
| 6 | Italy | 27 | 3.85 | 403 | 14.93 |
| 7 | Japan | 23 | 3.28 | 389 | 16.91 |
| 8 | United Kingdom | 21 | 2.99 | 1099 | 52.33 |
| 9 | South Korea | 19 | 2.71 | 296 | 15.58 |
| 10 | Canada | 17 | 2.42 | 516 | 30.35 |

TAP, transversus abdominis plane block.

**Table S2.** Top 10 institutions contributing to TAP block research from 2007 to 2024.

| Rank | Institution | Country | Number of publications | Total citations | Average citation per publication |
| --- | --- | --- | --- | --- | --- |
| 1 | University of Copenhagen | Denmark | 11 | 403 | 36.64 |
| 2 | Ain Shams University | Egypt | 11 | 51 | 4.64 |
| 3 | University of Minnesota | United States | 9 | 242 | 26.89 |
| 4 | Assiut University | Egypt | 9 | 49 | 5.44 |
| 5 | University Health Network | Canada | 9 | 76 | 8.44 |
| 6 | Harvard Medical School | United States | 8 | 128 | 16 |
| 7 | Cairo University | Egypt | 8 | 66 | 8.25 |
| 8 | University of Toronto | Canada | 7 | 275 | 39.29 |
| 9 | Washington University | United States | 7 | 114 | 16.29 |
| 10 | Kyungpook National University | South Korea | 7 | 109 | 15.57 |

TAP, transversus abdominis plane block.

**Table S3.** Top 10 journals contributing to TAP block research from 2007 to 2024.

| Rank | Journal title | Number of publications | Impact factor (2024) | Journal citation report quartile (2024) | Research area |
| --- | --- | --- | --- | --- | --- |
| 1 | Surgical Endoscopy and Other Interventional Techniques | 32 | 2.4 | Q2 | Surgical Endoscopy |
| 2 | Bmc Anesthesiology | 24 | 2.3 | Q2 | Anesthesiology |
| 3 | Anesthesia & Analgesia | 19 | 4.6 | Q1 | Anesthesiology |
| 4 | Indian Journal of Anaesthesia | 19 | 2.9 | Q1 | Anesthesiology |
| 5 | Journal of Clinical Anesthesia | 19 | 5 | Q1 | Anesthesiology |
| 6 | Regional Anesthesia and Pain Medicine | 17 | 5.1 | Q1 | Anesthesiology |
| 7 | Cureus Journal of Medical Science | 13 | 1 | Q3 | Medicine |
| 8 | Journal of Clinical and Diagnostic Research | 13 | 0.2 | Q4 | Medicine |
| 9 | Journal of Pain Research | 13 | 2.5 | Q2 | Clinical Neurology |
| 10 | Journal of Anaesthesiology Clinical Pharmacology | 12 | 1.5 | Q3 | Anesthesiology |

TAP, transversus abdominis plane block.

**Table S4.** Top 10 cited references in TAP block research from 2007 to 2024.

| Rank | Title | Institution | First author (Date of publication) | Journal | Citations | PMID/  DOI |
| --- | --- | --- | --- | --- | --- | --- |
| 1 | Quadratus lumborum block versus transversus abdominis plane block for postoperative pain after cesarean delivery: a randomized controlled trial. | Corniche Hospital, United Arab Emirates. | Rafael Blanco (2016) | Regional Anesthesia and Pain Medicine | 67 | PMID: 27755488  DOI: 10.1097/AAP.0000000000000495 |
| 2 | Studies on the spread of local anesthetic solution in transversus abdominis plane blocks. | Galway University Hospitals, Ireland. | J Carney (2011) | Anaesthesia | 58 | PMID: 21851346  DOI: 10.1111/j.1365-2044.2011.06855.x |
| 3 | Comparison between ultrasound-guided transversus abdominis plane and conventional ilioinguinal/iliohypogastric nerve blocks for day-case open inguinal hernia repair. | Polyclinique Sévigné, France. | C Aveline (2011) | British Journal of Anaesthesia | 52 | PMID: 21177284  DOI: 10.1093/bja/aeq363 |
| 4 | Comparison of analgesic efficacy of subcostal transversus abdominis plane blocks with epidural analgesia following upper abdominal surgery. | University Hospitals of Leicester NHS Trust, UK. | G Niraj (2011) | Anaesthesia | 49 | PMID: 21457153  DOI: 10.1111/j.1365-2044.2011.06700.x |
| 5 | Ultrasound-guided continuous oblique subcostal transversus abdominis plane blockade: description of anatomy and clinical technique. | Northeast Health Wangaratta, Australia. | Peter D Hebbard (2010) | Regional Anesthesia and Pain Medicine | 47 | PMID: 20830871  DOI: 10.1097/aap.0b013e3181e66702 |
| 6 | Plasma ropivacaine concentrations after ultrasound-guided transversus abdominis plane block. | Royal Women's Hospital, Australia. | J D Griffiths (2010) | British Journal of Anaesthesia | 45 | PMID: 20861094  DOI: 10.1093/bja/aeq255 |
| 7 | Addition of dexmedetomidine to bupivacaine in transversus abdominis plane block potentiates postoperative pain relief among abdominal hysterectomy patients: A prospective randomized controlled trial. | King Abdulaziz University, Egypt. | Waleed A Almarakbi (2014) | Saudi Journal of Anaesthesia | 44 | PMID: 24843325  DOI: 10.4103/1658-354X.130683 |
| 8 | Efficacy of ultrasound-guided transversus abdominis plane block after laparoscopic bariatric surgery: a double-blind, randomized, controlled study. | Max Super Speciality Hospital, India. | Aparna Sinha (2013) | Obesity Surgery | 42 | PMID: 23361468  DOI: 10.1007/s11695-012-0819-5 |
| 9 | The analgesic efficacy of subarachnoid morphine in comparison with ultrasound-guided transversus abdominis plane block after cesarean delivery: a randomized controlled trial. | American University of Beirut-Medical Center, Lebanon. | Ghassan E Kanazi (2010) | Anesthesia & Analgesia | 40 | PMID: 20488929  DOI: 10.1213/ANE.0b013e3181e30b9f |
| 10 | Distribution patterns, dermatomal anesthesia, and ropivacaine serum concentrations after bilateral dual transversus abdominis plane block. | Copenhagen University Hospital, Denmark. | Jens Børglum (2012) | Regional Anesthesia and Pain Medicine | 35 | PMID: 22476239  DOI: 10.1097/AAP.0b013e31824c20a9 |

TAP, transversus abdominis plane block.
